# Exploring Physical Inactivity in Diabetic Adults: A Study From a Rural Health and Training Centre

**DOI:** 10.7759/cureus.92186

**Published:** 2025-09-12

**Authors:** Sugesh Chandran V, Pankaj B Shah

**Affiliations:** 1 Community Medicine, Sri Ramachandra Institute of Higher Education and Research, Chennai, IND

**Keywords:** diabetes, gpaq, non-communicable disease, physical activity, who

## Abstract

Background

Diabetes mellitus (DM) is one of the most prevalent non-communicable diseases (NCDs) and one of the leading causes of mortality in many developing countries, such as India. For patients with diabetes, regular physical activity (PA) can improve blood glucose levels and help prevent further complications.

Objective

The main objective of this study is to estimate the prevalence of physical inactivity and its associated risk factors among diabetic people, aged 30 to 60 years, attending the NCD clinic in the Rural Health and Training Centre (RHTC) of a medical college.

Material and methods

This cross-sectional study was conducted in an RHTC of a medical college in Chennai to estimate the prevalence of physical inactivity and its associated risk factors among diabetic people aged 30 to 60 years. IEC permission was obtained, and informed consent was taken from the participants. Data were collected after interviewing 157 participants using a semi-structured questionnaire. PA levels were assessed using the World Health Organization’s Global Physical Activity Questionnaire (WHO-GPAQ).

Results

The interview was conducted among 157 patients; the majority of them were female, literate, married, and unemployed. The prevalence of physical inactivity was observed in 58 participants (36.9%). Most participants had only a moderate level of PA. Older age, unemployment, and low socio-economic status showed significant differences in physical inactivity levels.

Conclusion

The study shows the PA levels among participants from a rural area. These findings can be used to incorporate new interventions to improve PA levels among rural diabetic patients.

## Introduction

Diabetes mellitus (DM) is a syndrome that poses a significant public health issue due to its increasing prevalence, morbidity, mortality, and treatment costs. The global population of individuals with diabetes is projected to increase by 42% by 2030 [1].

According to the International Diabetes Federation (IDF) Diabetes Atlas, 2025 edition, an estimated 629 million adults worldwide are living with diabetes, and this number is projected to reach 643 million by 2030 and 784 million by 2045 [2]. Low- and middle-income nations are anticipated to account for the majority of this rise, with China and India alone contributing 163.5 million individuals with diabetes worldwide [3].

Inadequate physical activity (PA) ranks among the top 10 global risk factors for mortality and is a significant contributor to non-communicable diseases (NCDs), including cardiovascular diseases, cancer, and diabetes [4]. PA offers substantial health advantages and aids in the prevention of NCDs [5]. It is defined as “any bodily movement produced by skeletal muscles that results in energy expenditure” [6].

The American Diabetes Association and the World Health Organization recommend that persons with diabetes engage in moderate-intensity exercise for at least 150-300 minutes, or strenuous exercise for 75-150 minutes, or an equivalent combination of the two, including aerobic activity, on a weekly basis [7].

Research conducted over the last few decades has shown that PA is crucial to the treatment of DM. It may help prevent long-term complications or delay the progression of existing ones [2]. However, despite its many advantages, PA remains neglected in self-care management. Most DM patients in developing countries do not engage in regular PA due to sedentary lifestyles, rapid urbanization, and sociodemographic disparities [2].

Glycaemic management is improved by mild to moderate physical exercise, as measured by glycated haemoglobin (HbA1c) and fasting blood glucose levels. Such positive changes in HbA1c may lower the risk of cardiovascular disease - the leading cause of death among diabetic patients - and help prevent long-term microvascular complications [8]. With an average follow-up of eight years, patients with diabetes who are physically inactive or completely sedentary have a three- to fourfold higher risk of dying from coronary heart disease [8].

In India, urban populations have access to reliable screening methods and anti-diabetic medications, whereas rural patients often lack these health benefits. This leads to an unequal distribution of healthcare services between urban and rural regions [9].

This study aims to focus on the PA levels among diabetic patients aged 30 to 60 years attending the NCD clinic of a Rural Health and Training Centre (RHTC) of a private medical college in Chennai, Tamil Nadu, India.

Objectives

The primary objective is to estimate the prevalence of physical inactivity (defined as <600 MET-min/week) among adults with type 2 diabetes, aged 30-60 years, attending the NCD clinic of an RHTC.

The secondary objective is to examine the association between PA levels and sociodemographic factors such as age, sex, socioeconomic status, and employment status.

## Materials and methods

Study design and sample size

This cross-sectional study was conducted in an RHTC of a tertiary medical college hospital in Chennai, India. The sample size for the study was calculated using a prevalence of 52.1% from a study conducted by Sharma et al., with a 95% confidence interval and 15% relative precision [10]. Based on these findings, the final sample size calculated for this study was 157. Participants who fulfilled the inclusion criteria and provided informed consent were enrolled consecutively until the required sample size of 157 was reached. This method corresponds to a consecutive sampling technique, a form of non-probability sampling. We recruited 157 diabetic patients attending the NCD clinic to estimate their PA levels during the study period, from April to July 2023.

Inclusion criteria

Participants included in the study were known cases of type 2 DM, aged 30-60 years, diagnosed before March 2023 (as per clinic records), attending the NCD clinic at the RHTC during the study period (April-July 2023), and willing to provide written informed consent.

Exclusion criteria

Patients with type 1 diabetes or gestational diabetes, pregnant women, those with acute medical illnesses or who were currently hospitalized, and patients with mobility-limiting conditions likely to affect PA measurement - such as recent fractures, severe osteoarthritis, advanced diabetic neuropathy, or any other musculoskeletal or neurological disability impairing usual activity - were excluded. Patients with cognitive impairment or psychiatric illness that limited their ability to understand the questionnaire or provide informed consent were also excluded.

Study tools

The data collection questionnaire comprised semi-structured inquiries regarding demographic details such as name, age, gender, address, education, occupation, socio-economic status (calculated using the Modified B.G. Prasad Scale [11]), family type, and diabetes duration (see Appendix). PA was assessed using the World Health Organization’s Global Physical Activity Questionnaire (GPAQ) version 2, a standardized and validated instrument available in English [12]. The primary investigator, who was trained in the use of the GPAQ and its scoring protocol, directly conducted all interviews with eligible participants during their clinic visits.

The primary outcome was physical inactivity, defined as less than 600 MET-minutes per week, measured using the WHO GPAQ. The activity categories were as follows: low (<600 MET-min/week), moderate (600-2999 MET-min/week), and high (≥3000 MET-min/week). The GPAQ captured activity in three domains: work-related activity, transport (commuting) activity, and leisure-time activity. Sedentary behaviour was recorded as daily sitting or reclining time, measured in minutes per day, as per GPAQ item P16 [12].

Ethics approval 

Approval from the Institutional Research Ethics Committee of Sri Ramachandra Institute of Higher Education and Research was obtained (REF: CSP-MED/23/MAR/85/54). After the study was explained, participants provided written informed consent. Following consent, the investigator conducted interviews using the standardized questionnaire, ensuring participant privacy throughout the procedure.

Data entry and analysis

The entire dataset was first entered into Microsoft Excel (Microsoft® Corp., Redmond, WA, USA). The metabolic equivalent of task (MET) values were computed in accordance with GPAQ criteria. Following current guidelines, MET values were used to evaluate PA for each individual. Total energy expenditure was calculated using GPAQ data, with four METs assigned to the duration of moderate activities and eight METs to the duration of vigorous activities. The categorical indicator was computed based on the total duration of PA in a typical week, considering both the number of days and the intensity of the activity. Individuals were considered active if, over the course of a week, they engaged in a minimum of 150 minutes of moderate-intensity PA, 75 minutes of vigorous-intensity activity, or an equivalent combination of both, resulting in at least 600 MET-minutes. Overall PA was quantified in MET-minutes per week and categorized as low (<600 MET-min/week), moderate (600-2999 MET-min/week), or vigorous (≥3000 MET-min/week) [12]. Data analysis was performed using the Chi-square test for categorical comparisons and ANOVA for mean MET-minute calculations, with SPSS for Windows, Version 16.0 (Released 2007; SPSS Inc., Chicago, IL, USA).

## Results

Table 1 shows the participants' socio-demographic details, which include age, sex, educational qualification, marital status, employment status, socioeconomic class, and family type. The mean age of the study participants was 50.68 years, with a standard deviation of 8.05. 

**Table 1:** Socio-demographic details of the participants

Background characteristics	N (%)
Age	
30 - 40	26 (16.6%)
41 - 50	42 (26.8%)
51 - 60	89 (56.7%)
Sex	
Male	63 (40.1%)
Female	94 (59.9%)
Educational qualification	
Illiterate	35 (22.3%)
Literate	122 (77.7%)
Marital status	
Married	144 (91.7%)
Unmarried	10 (6.4%)
Separated	3 (1.9%)
Employment status	
Unemployed	91 (58%)
Employed	66 (42%)
Socio-economic status	
Upper class	27 (17.2%)
Upper middle class	24 (15.3%)
Middle class	33 (21%)
Lower middle class	42 (26.8%)
Lower class	31 (19.7%)
Family type	
Nuclear	82 (52.2%)
Joint	75 (47.8%)

Table 2 shows the PA levels among the study participants. Out of 157 subjects, 58 (36.9%) had low PA, and only 15 (9.6%) engaged in high PA. Males were more physically active than females in this study. 

**Table 2:** Level of physical activity attained by the study participants measured in MET min/week MET, metabolic equivalent of task

Level of physical activity	Frequency (%)		Total (%) (n = 157)
	Male (n = 63)	Female (n = 94)	
Low (<600 MET min/week)	20 (31.7)	38 (40.4)	58 (36.9)
Moderate (600-2999 MET min/week)	34 (54)	50 (53.2)	84 (53.5)
High (≥3000 MET min/week)	9 (14.3)	6 (6.4)	15 (9.6)

Table 3 explores the association between socio-demographic variables and levels of PA. A significant difference was found between age and PA levels; participants who were under 45 years of age were more physically active than those over 45. Similarly, socioeconomic status showed a statistically significant difference in PA levels, with participants from the upper-income class being more active than those from the lower-income class. Employment status also showed a significant association, as employed participants were more physically active than those who were unemployed. However, education level, duration of diabetes, and marital status did not show any significant differences in PA levels. 

**Table 3:** Relationship between demographic variables and level of physical activity among study participants

Demographic variables	Low	Moderate	High	p-value
	N = 58 (36.9)	N = 84 (53.5)	N = 15 (9.6)	
Age				
≥45 Years	50 (43.9%)	58 (50.9%)	6 (5.3%)	0.001
<45 Years	8 (18.6%)	26 (60.5%)	9 (20.9%)	
Socio-economic class				
Upper	6 (22.2)	14 (51.9)	7 (25.9)	0.012
Upper middle	5 (20.8)	17 (70.8)	2 (8.3)	
Middle	12 (36.4)	19 (57.6)	2 (6.1)	
Lower middle	17 (40.5)	22 (52.4)	3 (7.1)	
Lower	18 (58.1)	12 (38.7)	1 (3.2)	
Education				
Illiterate	16 (45.7)	18 (51.4)	1 (2.9)	0.213
Literate	42 (34.4)	66 (54.1)	14 (11.5)	
Employment status				
Employed	15 (22.7)	37 (56.1)	14 (21.2)	0.001
Unemployed	43 (47.3)	47 (41.6)	1 (1.1)	
Duration of diabetes				
=5 years	26 (31.0)	47 (56.0)	11 (13.1)	0.115
>5 years	32 (43.8)	37 (50.7)	4 (5.5)	
Marital status				
Married	54 (37.5)	77 (53.5)	13 (9.0)	0.511
Unmarried	2 (20.0)	6 (60.0)	2 (20.0)	
Separated	2 (66.7)	1 (33.3)	0 (0.0)	

## Discussion

This study was conducted to estimate the prevalence of physical inactivity among diabetic patients aged 30 to 60 years, attending the NCD clinic of an RHTC of a medical college. Out of 157 interviewed participants, the majority were females (59.9%). This is consistent with studies conducted by Fattahi et al. among type 2 diabetes patients in Hamadan, Iran [13]; Palermo and Sandoval at the Philippine General Hospital [14]; and Mwimo et al. in Northern Tanzania [15]. The majority of the study participants were between the ages of 41 and 60 years. This is an important age group because of its productivity and its major economic contribution to the family. This finding is consistent with studies conducted in Northwest Ethiopia [2], Iran [13], and India [8]. The present study shows that males are more physically active than females. This is consistent with findings from previous studies, which also show that the level of PA in males is higher than in females [16-18].

Nearly 23% of the study participants were illiterates, which was similar to the study conducted by Enyew et al. [19], which shows 22.8% illiteracy among the study subjects [2], and much higher compared to the study in Marappadi, which shows only 11.76% illiteracy among the participants [5]. Most of the study participants were married in this study, and this was similar to many other studies [5,8,13-15]. The Modified BG Prasad classification was used to calculate the socioeconomic class, and it was found that the participants were equally distributed across all classes.

The present study shows that 58 (36.9%) of the participants had a low level of PA, 84 (53.5%) had moderate PA, and only 15 (9.6%) had a high level of PA. This is consistent with a study conducted by Fattahi et al., which revealed that most of the participants (57.5%) had a moderate level of PA [13]. A cross-sectional study conducted by Patil et al. in the Indira Gandhi Government Medical College shows that 27% of the participants had a moderate level of PA [20]. In a study by Çolak et al. in Turkey, 39.5% had low, 51.9% had moderate, and 8.5% had a high level of PA [21]. A cross-sectional study conducted by Palermo and Sandoval among type 2 DM patients shows that 68.9% of the participants had moderate to high levels of PA [14].

In the present study, age, socioeconomic status, and employment status had a significant difference in the levels of PA. In our study, it was found that there was no significant difference between marital status and physical inactivity. Contrary to this finding, a study among 320 diabetic patients by Fattahi et al. shows that PA had a statistically significant association with age, education, occupation, and marital status. Fattahi et al.'s study shows a significant difference between age, education, occupation, and marital status in relation to PA [13]. Even though this study did not show any statistical difference between education and PA, education is considered an indicator of participation in, and duration of, PAs according to multiple other studies [22-24].

According to the WHO’s recommendation, a person has to do at least 150 minutes of moderate to vigorous-intensity PA per week to maintain their physical health at an optimum level. In India, nearly half of the population is currently not following this recommendation, leading to an increase in the incidence of NCDs. More people are obtaining their PA levels only from their employment domain. This study also shows that employed people are more physically active than the unemployed. This is similar to study findings from China and Vietnam, where most of the participants were obtaining their PA only from their work [25,26]. This highlights the necessity of raising PA awareness in India and providing resources for people to participate in leisure PA in both urban and rural locations.

Strengths

The major strength of the study is that it focuses on an important and economically productive age group of 30 to 60 years in a rural area with DM, where data is limited. The focus on rural areas helps fill the gap in research and also helps policymakers make appropriate community-based practical implications.

Limitations

The findings of the study may not be applicable to the entire diabetes population, as it was conducted only among outpatients. Being a cross-sectional study, causal relationships between socio-demographic factors and physical inactivity cannot be established. PA was self-reported, introducing potential recall and social desirability biases.

## Conclusions

The study found that the majority of the participants had only a moderate or low level of PA. Factors such as increasing age, unemployment, and lower socio-economic class showed a statistically significant association with lower PA. Male participants were found to be more physically active than females. These findings emphasize the need for increasing awareness of PA, especially among older people, women, and those from low socio-economic backgrounds, since increasing PA levels among diabetes patients can help with better control of diabetes and improve overall quality of life.

## Appendices

Annexure

Interview schedule

1. Name:

2. Sex:

3. Age:

4. Address:

5. Education:

6. Occupation:

7. Socio-economic status:

8. Type of family:

9. No. of individuals in the family:

10. Personal h/o:

11. Associated medical problems:
